# Mechanisms linking multi-year La Niña with preceding strong El Niño

**DOI:** 10.1038/s41598-021-96056-6

**Published:** 2021-08-25

**Authors:** Tomoki Iwakiri, Masahiro Watanabe

**Affiliations:** Atmosphere and Ocean Research Institute, The University of Tokyo, 5-1-5 Kashiwanoha, Kashiwa, Chiba 277-8568 Japan

**Keywords:** Atmospheric science, Physical oceanography

## Abstract

El Niño-Southern Oscillation (ENSO), characterized by anomalous sea surface temperature in the central-eastern equatorial Pacific, is a dominant interannual variability, impacting worldwide weather and socioeconomics. The ENSO cycle contains irregularity, in which La Niña often persists for more than two years, called “multi-year La Niña”. Observational records show that multi-year La Niña tends to accompany strong El Niño in the preceding year, but their physical linkage remains unclear. Here we show using reanalysis data that a strong El Niño excites atmospheric conditions that favor the generation of multi-year La Niña in subsequent years. Easterly wind anomalies along the northern off-equatorial Pacific during the decay phase of the strong El Niño are found crucial as they act to discharge ocean heat content (OHC) via an anomalous northward Ekman transport. The negative OHC anomaly is large enough to be restored by a single La Niña and, therefore, causes another La Niña to occur in the second year. Furthermore, analyses of the Coupled Model Intercomparison Project Phase 6 (CMIP6) climate models support the abovementioned mechanisms and indicate that the occurrence frequencies of multi-year La Niña and strong El Niño are highly correlated.

## Introduction

The El Niño-Southern Oscillation (ENSO) is a quasi-periodic variability with irregularity in several aspects such as amplitude and transition, and the life cycle is known to be locked with seasonal march: growing in boreal spring and summer, maturing in winter, and decaying in the following spring^1–3^. The oscillatory nature of ENSO has been well explained by the conceptual theory^4–8^. The recharge oscillator paradigm has been established based on the fact that equatorial Pacific warm water volume anomalies precede the ENSO sea surface temperature (SST) anomaly^6,7,9^. According to the theory, transition from La Niña to El Niño occurs such that the intensified easterly trade winds associated with the La Niña SST anomaly act to recharge warm subsurface water from the off-equator to the equatorial strip via a slow Sverdrup adjustment. The deepened thermocline weakens the cooling effect due to mean upwelling in the eastern equatorial Pacific, which eventually causes the El Niño SST anomaly to grow: the process referred to as the thermocline feedback. The slow ocean dynamical process has been verified using a phase relationship between the observed warm water volume anomaly in the equatorial Pacific and the ENSO SST anomaly^10–13^.

In reality, the temporal behavior of ENSO is highly complex^14^. Unlike solutions in the recharge oscillator equations, observations show that ENSO has a strong transition asymmetry; El Niño terminates rapidly but La Niña often lasts more than two years, the latter referred to as multi-year La Niña^15^. In particular, extremely strong El Niño events tend to terminate and transition to La Niña within one year. When strong El Niño decays from winter to spring, equatorial westerly wind anomalies are known to shift southward^16^, causing equatorial thermocline shoaling and contributing to the rapid termination of strong El Niño^17–19^. This atmospheric pattern with southward-shifted westerly anomalies is induced by a nonlinear coupling between ENSO’s interannual frequency and the annual cycle in the western Pacific, known as a combination mode (C-mode)^20–22^.

The multi-year persistency of La Niña has been investigated using observations and climate model simulations. A key process for the ENSO phase transition is a delayed negative thermocline feedback (equivalent to the recharge-discharge process), which is weak for La Niña and may, therefore, explain the longer duration of La Niña than El Niño^23^. A report also states that the SST anomaly pattern has a wide meridional structure when La Niña persists for more than two years^24^. Because the recharge after the peak of La Niña is ineffective with a wide meridional pattern of the wind stress curl, the spatial difference in the SST anomaly may contribute to the multi-year persistency of La Niña. In addition, remote influences from the Atlantic and Indian Ocean may play a role in the multi-year persistence of La Niña that was predicted two years ahead in a climate model^25^.

A common suggestion from previous studies is that a multi-year La Niña tends to occur after a strong El Niño. A recent study shows that the duration of La Niña is strongly influenced by the amplitude of the preceding El Niño in both observations and a long climate model simulation, presumably due to a large initial discharge^26^. However, the physical link between strong El Niño and the subsequent multi-year persistence of La Niña has not been comprehensively examined using ocean subsurface reanalysis data. In particular, a question of how extremely strong El Niño that decays rapidly^21^ can influence the subsequent occurrence of multi-year La Niña is unclear.

In this study, we address the following questions. What process distinguishes multi-year La Niña from single-year La Niña? Is there a dynamical connection between strong El Niño and a subsequent multi-year La Niña? By answering these questions, we attempt to identify triggers for multi-year La Niña. In addition to the observational analyses, we investigate the connection between strong El Niño and multi-year La Niña in 23 CMIP6 models and demonstrate that the observed physical linkage is consistently seen in the climate model ensemble. There are several reports that a multi-year La Niña in the first and second years has distinct impacts on the atmospheric circulation over North America and East Asia^15,27,28^. Although ENSO forecasting for more than one year is still challenging^29,30^, deepening our understanding of the mechanism of multi-year La Niña and its connection to a preceding strong El Niño would help improve seasonal and long-term climate prediction.

## Results

### Time evolution of multi-year La Niña

Using observed Niño 3.4 SST anomalies (hereafter N3.4) during 1961–2016, we identified ten La Niña events, out of which six and four, respectively, are categorized into multi-year and single-year events (Fig. S1). We define the period when La Niña develops in the first year as Year (0). By taking composites for these events of N3.4 and the equatorial Pacific ocean heat content (OHC) (OHC_eq_: 120° E–60° W, 5.5° S–5.5° N, surface-500 m), the latter measuring the ENSO-related anomalies in the warm water volume, trajectories associated with the multi-year and single-year La Niñas are obtained in the phase space (Fig. 1a).

**﻿Figure 1:**
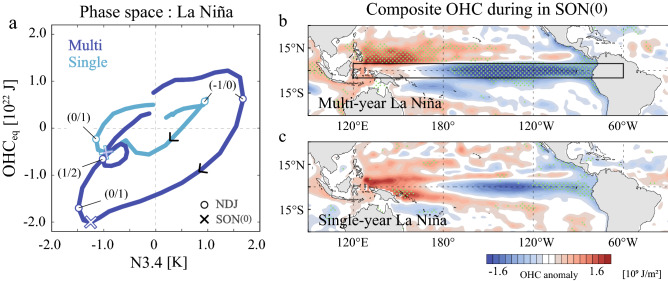
Comparison of the phase transition between multi-year and single-year La Niña. (**a**) Phase space diagram for observed composite multi-year (blue) and single-year (light blue) La Niña plotted for the periods preceding El Niño to the termination of La Niña. The horizontal axis is N3.4 and the vertical axis is the OHC_eq_ anomaly. The trajectories are smoothed with a three-month running-mean. Circles denote NDJ for each year and crosses denote SON(0). (**b**, **c**) Multi-year and single-year La Niña composite maps of OHC anomalies integrated from the surface to 500 m during SON(0), respectively. Dots indicate anomalies statistically significant at the 95% confidence level. The box in (**b**) represents the region to calculate OHC_eq_. (Maps are generated by visualize module PyNGL with Python Programming Language version 3.7; https://www.pyngl.ucar.edu/).

The composite-mean trajectories are akin to the limit cycle^6,10^, showing that La Niña in Year (0) follows El Niño in Year (-1), both of which reach their peak in boreal winter (November–December–January: NDJ, circles in Fig. 1a). Four out of six multi-year La Niña events accompany strong El Niños (defined by the ONDJF-mean N3.4 exceeding the 1.5 standard deviation) in the preceding year, whereas all El Niño events before a single-year La Niña have a moderate amplitude (Fig. S1). Consistent with the recharge theory, the OHC_eq_ anomaly leads N3.4 by 2–3 months and peaks in September–October–November (SON) before the mature phase of La Niña. While the positive OHC_eq_ anomaly during SON(-1) is comparable in magnitude between the two composites, the negative OHC_eq_ anomaly in SON(0) preceding multi-year La Niña is four times as large as that of single-year La Niña. Despite the large difference in the OHC_eq_ anomaly, the negative N3.4 in NDJ(0/1) in the multi-year La Niña composite is only 1.3 times larger than that in the single-year composite probably due to a strong asymmetry in the thermocline feedback and nonlinear dynamical heating that suppress La Niña but enhance El Niño^10,31,32^. This contrast in magnitude between the SST and OHC_eq_ anomalies is also an important feature of multi-year La Niña and may contribute to the recurrence of La Niña as described latter.

After NDJ(0/1), the negative OHC_eq_ anomaly eventually turns positive in the single-year composite, leading to the termination of La Niña. In contrast, the OHC_eq_ anomaly in the multi-year composite decays but remains negative until the following year, resulting in a re-intensification of subsequent La Niña in NDJ(1/2). The time evolution shown in Fig. 1a indicates that the recurrence of La Niña is consistent with the linear recharge oscillator theory except for an extraordinary negative OHC_eq_ anomaly in NDJ(0/1) that is not restored within a year and therefore a crucial harbinger of multi-year La Niña^26^.

The spatial distribution of composite OHC anomalies in its peak season, SON(0), is shown in Fig. 1b,c. The multi-year composite is characterized by a contrast between large negative OHC anomalies in the equatorial Pacific and positive anomalies in the western-central Pacific at approximately 5°–15° N, suggesting that the former was induced by a mass exchange between the equatorial strip and northern off-equator. Such an equatorial asymmetry is not observed in the single-year composite, which indicates weak negative OHC anomalies in the central-eastern equatorial Pacific and positive anomalies in the tropical western Pacific.

### Physical link between multi-year La Niña and preceding strong El Niño

**Figure 2:**
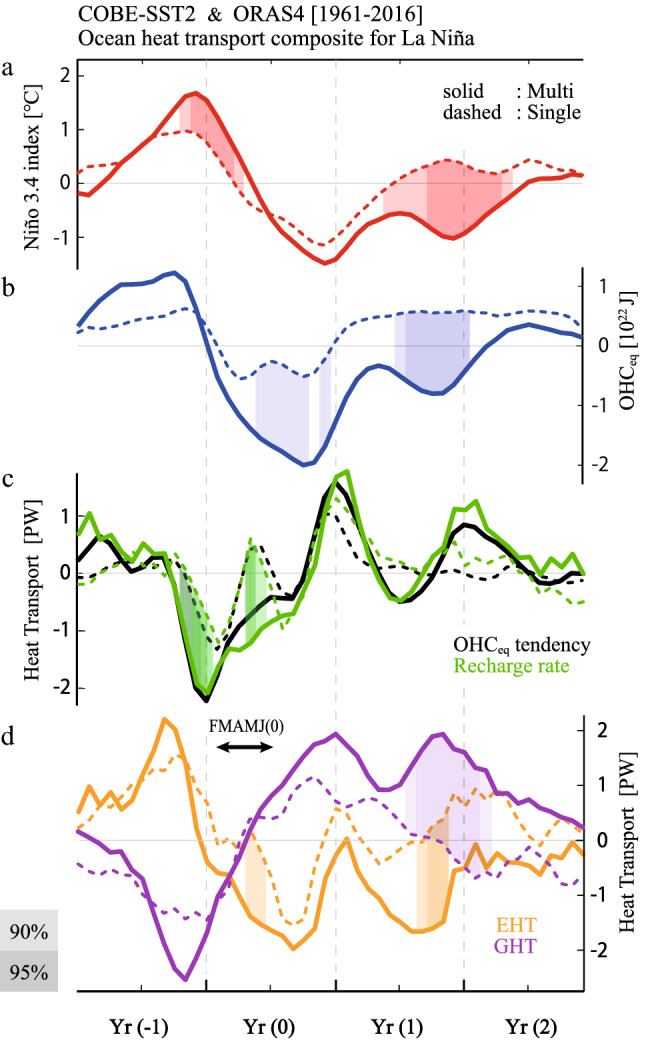
Time evolution of multi-year and single-year La Niña and associated recharge processes. Time series of composited multi-year (solid curves) and single-year (dashed curves) La Niña events for (**a**) N3.4 and (**b**) OHC_eq_ anomalies; (**c**) OHC_eq_ tendency (black) and its reconstruction by the recharge rate (green) (Methods); and (**d**) contribution to the recharge rate by the geostrophic term (GHT, purple) and the Ekman term (EHT, orange). Shadings indicate the difference between multi-year and single-year composites statistically significant at the 90 and 95% confidence levels. The time series are smoothed with a three-month running mean after the individual budget term was calculated.

A significant difference between multi-year and single-year La Niña events is clearly observed in the composite time series of N3.4 and OHC_eq_ anomalies (Fig. 2a,b). To clarify processes responsible for the strong discharge of warm water during the growth phase of multi-year La Niña, heat budget analysis was performed on OHC_eq_ using ocean reanalysis datasets (Methods). While our conclusion depends little on the choice of the dataset (Fig. S2), we present the result based on ORAS4 in Fig. 2b-d because the errors in the heat budget were the smallest among the four datasets.

A large negative OHC_eq_ anomaly in SON(0) for the multi-year La Niña composite occurs because of a large negative OHC_eq_ tendency during the first half of Year (0), i.e., from February to June, denoted as FMAMJ(0) (black solid curve in Fig. 2c). For both multi-year and single-year La Niña composites, the OHC_eq_ tendency is reproduced well by the aggregated OHC_eq_ budgets (shading in Fig. S2a,e), which can then be decomposed into individual terms (zonal and meridional advection terms due to geostrophic and Ekman currents, heat exchange at the bottom of the subsurface, and net heat flux at the surface). The sum of the geostrophic and Ekman terms (i.e., Sverdrup heat transport) is called the recharge rate, which explains most of the OHC_eq_ tendency (green curves in Fig. 2c). Unlike the linear recharge oscillator theory, the composited recharge rate indicates a persistent large negative anomaly during FMAMJ(0) when the N3.4 signal is weak.

The decomposition of the recharge rate into components of surface Ekman current heat transport (EHT) and geostrophic current heat transport (GHT) is shown in Fig. 2d. On one hand, the GHT during the peak period of preceding El Niño shows a large negative anomaly in the multi-year composite compared to the single-year composite; however, they are actually similar after normalized with N3.4 (Table S1). The magnitude of GHT is inversely proportional to N3.4 throughout the period and this implies that the GHT-induced discharge is not a primary factor for generating the OHC_eq_ difference between multi-year and single-year La Niña events. On the other hand, EHT in the multi-year composite becomes negative in FMAMJ(0) and contributes to the large negative recharge rate (i.e., discharge); this feature is not observed in the single-year composite (Fig. 2d). The composite heat transport at the northern and southern boundaries indicates that the strong discharge for multi-year La Niña in FMAMJ(0) is attributed to EHT across the northern boundary, which is slightly weakened by the heat imported across the southern boundary (Fig. S3).

**Figure 3:**
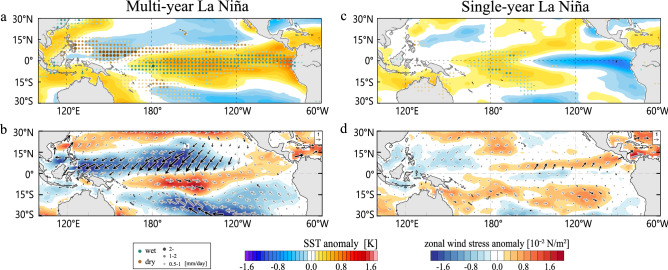
Multi-year and single-year La Niña composite maps in FMAMJ(0). (**a**) Anomalous SST (shading) and precipitation (dots) and (**b**) surface zonal wind stress (shading) and wind stress vector (unit in 10^−2^ N/m^2^) for the multi-year La Niña composite. (**c**, **d**) As in (**a**, **b**) but for the single-year La Niña composite. Vectors in black indicate anomalies statistically significant at the 95% confidence level. (Maps are generated by visualize module PyNGL with Python Programming Language version 3.7; https://www.pyngl.ucar.edu/).

**Figure 4:**
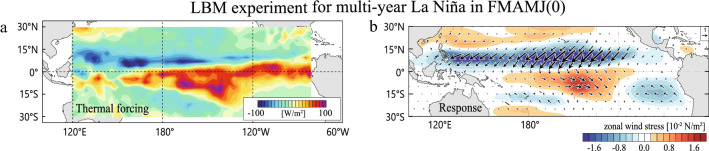
Diagnosing thermally forced steady linear response during FMAMJ(0) for multi-year La Niña. (**a**) Vertically integrated prescribed thermal forcing in multi-year La Niña composite (six events mean). Forcing is assumed to be zero outside of the tropical Pacific. (**b**) Steady response in the LBM (Methods) of surface zonal wind stress (shading) and wind stresses (vector: unit in 10^−2^ N/m^2^) forced by the diabatic heating shown in (**a**). (Maps are generated by visualize module PyNGL with Python Programming Language version 3.7; https://www.pyngl.ucar.edu/).

During FMAMJ(0) corresponding to the transition period from El Niño to La Niña, composite anomalies of SST, precipitation, and surface wind stresses show different patterns between multi-year and single-year La Niña events (Fig. 3). The El Niño SST pattern persists in the multi-year La Niña composite but it has turned to a weak La Niña pattern in the single-year composite (Fig. 3a,c) because strong El Niño tend to last several more months compared to weak El Niño^26^. In the multi-year composite map, anomalous positive precipitation is found to the south of the equator, while negative precipitation anomalies appear along the northern off-equator. This southward shift of the precipitation anomaly pattern has been identified during the decay phase of strong El Niño^20^. Consistently, eastward wind stress anomalies are present over the south of the equator and westward anomalies dominate over the northern off-equator, the latter being responsible for the strong EHT-induced discharge (Fig. 3b). We confirmed that the anomalous cross-equatorial wind stresses can be dynamically excited by diabatic heating anomalies associated with the southward-shifted precipitation anomaly pattern shown in Fig. 3a (Fig. 4).

Overall, anomaly patterns in precipitation and wind stresses in the multi-year composite are consistent with the equatorial asymmetric distribution of OHC anomalies during SON(0) (Fig. 1b). These atmospheric anomalies are indicative of the C-mode that appears during strong El Niño^17,22^ (Fig. S4, see also supplementary Methods). The single-year composite maps lack anomalous zonal wind stress over the off-equator as expected from symmetric weak precipitation anomalies around the equator (Fig. 3c,d). While the EHT-discharge in Year (0) of the first year can be explained by the C-mode associated with strong El Niño, there is another EHT-discharge contributing to La Niña in Year (1) (Fig. 2d). There is a possibility that processes that cause the ENSO amplitude asymmetry also play a role for persistency of the La Niña condition.

Because two out of six multi-year La Niña events do not accompany strong El Niño, the preceding strong El Niño would not be a unique cause of multi-year La Niña. Yet, the importance of the easterly wind stress anomaly over the northern off-equator for multi-year La Niña is robust. We classified multi-year La Niña and strong El Niño into three categories based on its phase transition. The first category, which explains majority of events, represents the transition from strong El Niño to multi-year La Niña (four events; 1972–75, 82–85, 97–90, 09–12) and the composite anomalies of this type show a quite similar result to the multi-year La Niña composite in Figs. 2 and 3 (Fig. S5). The second category consists of two multi-year La Niña events (1970–72 and 2007–09), which are preceded by moderate El Niño (Fig. S6). Compared to the first category, the C-mode is not identified because of weak SST and precipitation anomalies. Nevertheless, the easterly wind stress anomaly occurring along the northern off-equator induces the EHT-discharge in Year (0) besides the EHT-discharge in Year (1) also strongly contributes to the recurrence of La Niña. The third category (1965–66 and 1991–92) represents the transition from strong El Niño to a neutral phase but not multi-year La Niña (Fig. S7). The composite anomalies for these two El Niño events show that the OHC_eq_ anomaly is very weak in Year (0/1). Consistently, easterly wind stress anomalies are absent along the northern off-equator during FMAMJ(0). The precipitation anomaly in this category is symmetric about the equator, and it cannot excite the northern off-equatorial easterly stress anomaly that is a part of the surface wind response asymmetric about the equator. These results confirm that the anomalous easterly winds and associated EHT-induced discharge in the northern off-equatorial latitudes are the key to link multi-year La Niña with a preceding El Niño, but they do not always occur in the decay phase of strong El Niño and can even be excited by other factors.

### Analysis of CMIP6 control simulations

To obtain a robust physical relationship between strong El Niño and multi-year La Niña, we repeated our analyses to 500-year long preindustrial control simulations using 23 Earth system models (ESMs) participating in CMIP6^33^ (Table S2). First, we evaluated the ability of ESMs to generate multi-year La Niña defined for each model having different ENSO amplitude (Methods). Fortunately, many models and thus the multi-model mean capture the observed time evolution of both single-year and multi-year La Niñas although some models generate very few multi-year events (Fig. S8 and Table S2).

**Figure 5:**
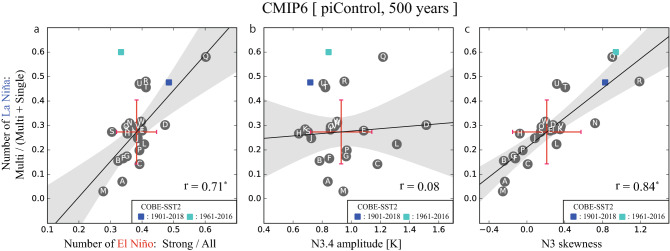
Relationship between the occurrence frequency of multi-year La Niña and the ENSO characteristics in CMIP6 pre-industrial control simulations. The vertical axis is a ratio of the number of multi-year La Niña against the total number of La Niña events. The horizontal axes are (**a**) a ratio of the number of strong El Niño against all El Niño events, (**b**) ENSO amplitude defined by one standard deviation of N3.4, and (**c**) ENSO amplitude asymmetry defined by skewness of the SST anomaly averaged in the Niño 3 region (90°–150° W, 5° S–5° N). Alphabetical labels indicate individual CMIP6 models shown in Supplementary Table S2, and blue dots show the observational references for two periods (1901–2018 and 1961–2016), The correlation coefficients among 23 models are shown at the bottom right and asterisks indicate statistically significant at the 95% confidence level. The red error bars denote one standard deviation range of the multi-model ensemble. The black line and grey shading represent the regression curve and its 95% confidence range, respectively.

The ratio of the number of multi-year events against the total number of La Niña in observations is 0.60 for 1961–2016 and 0.48 for 1901–2018, but it varies across models, with the multi-model mean and the inter-model standard deviation are 0.27 ± 0.13 (Fig. 5a). On average, ESMs suffer from a bias of too few occurrence frequency of multi-year La Niña; however, some models capture the observed frequency of multi-year events. As expected, a significant positive correlation ($r = 0.71$) is found between the occurrence frequencies of multi-year La Niñas and those of strong El Niños in CMIP6 models. This indicates that multi-year La Niña tends to occur more often in a model that generates many strong El Niños, supporting a physical link between them. The more frequent occurrence of multi-year La Niña does not necessarily correspond to the large ENSO amplitude, and in fact, there is negligible correlation between the ENSO amplitude and the occurrence frequency of multi-year La Niña (Fig. 5b). On the contrary, the ENSO amplitude asymmetry is highly proportional to the occurrence frequency of multi-year La Niña ($r = 0.84$, Fig. 5c), supporting our assumption that the ENSO amplitude asymmetry is also at work for generating multi-year La Niña.

**Figure 6:**
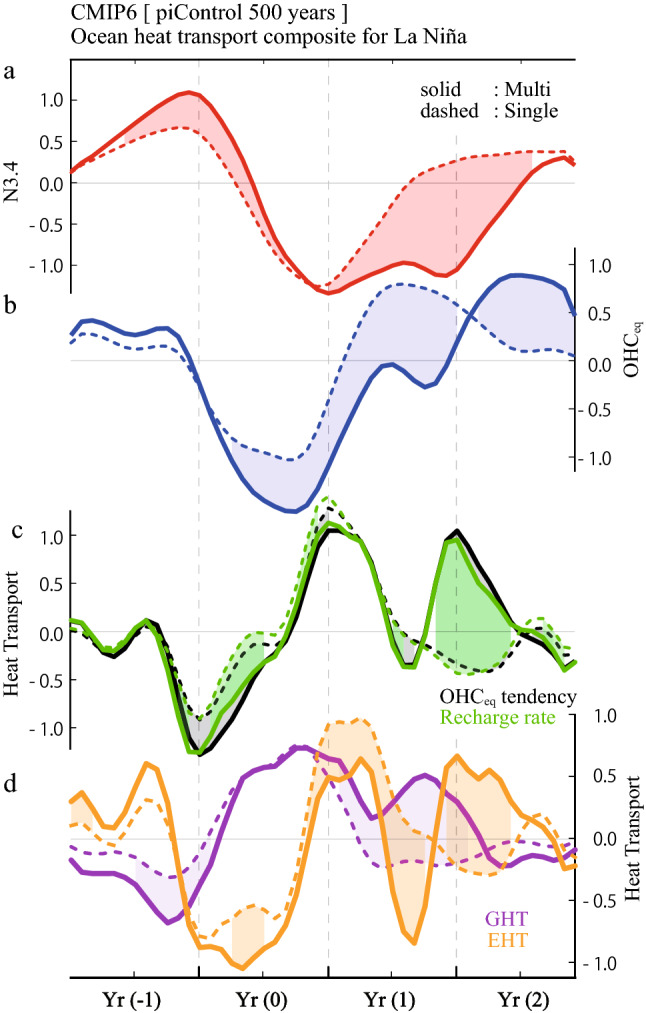
Time evolution of multi-year and single-year La Niña in CMIP6 models. As in Fig. 2 but for the results based on 23 CMIP6 control simulations, which contain 619 and 1796 multi-year and single-year La Niña events in total, respectively. Shadings indicate the difference between multi-year and single-year composites statistically significant at the 95% confidence levels. All indices are normalized.

We conducted heat budget analyses to the composite anomalies for multi-year and single-year La Niña events identified in CMIP6 models (Fig. 6). Time evolution of N3.4 and OHC_eq_ anomalies capture the observational features (Fig. 6a,b). Although negative OHC_eq_ anomaly during Year (1) of multi-year composite is weak in CMIP6 compared to observations, negative N3.4 continues to the next year because the OHC_eq_ anomaly does not change the sign. Unlike observation that contains less than ten events, the combined analysis to 23 control simulations is based on roughly 100 times more samples, leading to the difference between multi-year and single-year La Niña composites significant throughout the cycle.

**Figure 7:**
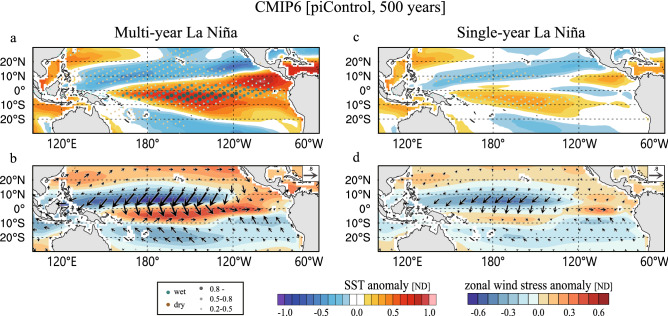
Multi-year and single-year La Niña composite maps in FMAMJ(0) in CMIP6 models. As in Fig. 3 but for the results based on 23 CMIP6 control simulations. All variables are normalized. (Maps are generated by visualize module PyNGL with Python Programming Language version 3.7; https://www.pyngl.ucar.edu/).

The OHC_eq_ tendency is approximated by the recharge rate, which shows a significant difference between multi-year and single-year La Niña events when El Niño turns to La Niña (Fig. 6c). Consistent with observations, decomposition of the recharge rate indicates the major role of EHT-discharge during FMAMJ(0) in generating a large negative OHC_eq_ anomaly for the multi-year events (Fig. 6d). Indeed, the FMAMJ(0) composite map for multi-year La Niña shows strong northeasterly wind stress anomalies over the northern off-equator, accompanied by northwesterly anomalies to the south, which we identified in observations the key for the EHT-discharge (Fig. 7a,b). The single-year La Niña composite also shows the northern off-equatorial easterly anomalies that are not seen in observations, but they are much weaker than the multi-year La Niña composite (Fig. 7c,d). Thus, the analysis of CMIP6 multi models supports the physical mechanism linking strong El Niño with subsequent multi-year La Niña.

## Discussion

**Figure 8:**
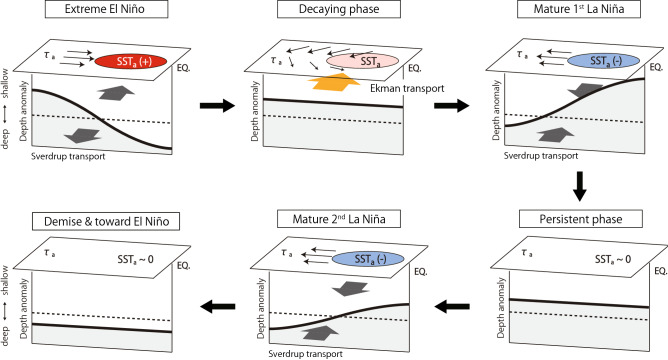
Schematic representation of multi-year La Niña triggered by strong El Niño. The time evolution is clockwise. Thick line indicates the thermocline depth anomaly from zero line (dashed line). Horizontal vector represents the surface wind stress anomaly. Red/blue shadings are the SST anomaly. Thick meridional arrows represent the geostrophic (gray) and Ekman (orange) heat transports. Figure is modified from a schematic diagram of the recharge oscillator^10^.

In this study, we examined the mechanisms of multi-year La Niña which occupies two third of La Niña events during the period 1961–2016. A physical link was identified between multi-year La Niña and strong canonical El Niño, of which the latter often accompanied in the preceding year. The essential mechanism for the transition from strong El Niño to multi-year La Niña, clarified using ocean reanalysis data sets and also supported by CMIP6 multi models, is explained by a modification of the recharge/discharge cycle as schematically illustrated in Fig. 8.

The pattern of surface wind stress anomalies, specifically easterly anomalies to the north and westerly anomalies to the south of the equator, occurs when strong El Niño decays. The off-equatorial easterly anomaly is effective in intensifying the northward Ekman heat transport, which acts as an extra term to discharge the warm water in the equatorial Pacific. This strong mass discharge of the upper ocean cannot be restored by a single La Niña and, therefore, causes another La Niña to occur in the second year. In essence, the intensified northward Ekman heat transport driven by surface easterly anomalies along the northern off-equator plays a central role in linking strong El Niño with subsequent multi-year La Niña. In the study, we focused on majority of events that show strong El Niño preceding multi-year La Niña, but there are a couple of multi-year La Niña events that occurred following moderate but not strong El Niño. Yet, those cases consistently indicate the Ekman discharge associated with easterly anomalies along the north off-equator . This supports our claim that the off-equatorial Ekman transport is the key for multi-year La Niña, but suggests that strong El Niño is not the unique cause.

Given that ENSO is positively skewed^31,32,34^, i.e., El Niño is stronger than La Niña, it is reasonable to doubt that multi-year La Niña is an apparent feature arising from the climatological mean shifted toward El Niño due to amplitude asymmetry. However, our results clearly show that multi-year La Niña is not a statistical artefact but a part of the intrinsic complex nature of ENSO. Previous studies have shown the contribution of anomalous wind stress curl causing GHT in the ENSO transition^12,24,35–37^, an active role of inter-basin interaction for the transition asymmetry^38^, and a meridional shift of westerly surface wind anomalies for the transition from strong El Niño to La Niña^16–18,39^. Although we do not exclude these processes during ENSO phase transition, we demonstrate that the anomalous Ekman heat transport is crucial to transition from strong El Niño to multi-year La Niña.

As the link between strong El Niño and multi-year La Niña is robust, the occurrence of multi-year La Niña may be predictable beyond the typical predictability of ENSO events^40^. If a coupled atmosphere–ocean model initialized with a strong El Niño condition can reproduce the wind stress pattern responsible for the anomalous Ekman heat transport, the subsequent two years, when a multi-year La Niña will occur, maybe predicted owing to the large memory in the ocean heat content^25,41^. Our multi-model analysis indicates relationship between the ENSO amplitude asymmetry and the occurrence frequency of multi-year La Niña. Although many models fail to reproduce the occurrence frequency of multi-year La Niña, improving reproducibility of the ENSO amplitude asymmetry may at the same time result in better reproducing multi-year La Niña.

## Methods

### Observations and reanalysis data

We used the observed monthly SST and precipitation datasets from COBE-SST2^42^ and PRECipitation REConstruction (PREC)^43^ of the National Oceanic and Atmospheric Administration, respectively. Four ocean reanalysis products that contain temperature, salinity, current velocity, and surface heat flux were also used: Ocean Reanalysis System 4 (ORAS4)^44^, Ocean Reanalysis System 5 and its backward extension (ORAS5)^45^, German contribution to the consortium for Estimating the Circulation and Climate of the Ocean system 2 (GECCO2)^46^, and Geophysical Fluid Dynamics Laboratory Ensemble Coupled Data Assimilation version 3.1 (GFDL-ECDAv3.1)^47^. For the atmospheric fields, we used combined data of ERA-40^48^ and ERA-Interim^49^, which were adopted for surface forcing in producing ORAS4. The analysis period was from January 1961 to December 2016, and de-trended anomalies were defined as deviations from monthly climatology calculated for the 30-year period from 1981 to 2010.

### CMIP6 multi models

We used preindustrial control simulations from 23 CMIP6 coupled global climate models^33^ (listed in Supplementary Table S2) to analyze monthly SST, precipitation, wind stresses, ocean temperature, salinity and meridional velocity (variable name: “tos”, “pr”, “tauu”, “tauv”, “to”, “so”, and “vo”). While each model was integrated for different periods, we used the first 500 years for the analysis. Atmospheric and surface variables are interpolated to a regular 1° × 1° longitude-latitude grid and oceanic variables to a 1.4° × 1.4° grid. De-trended anomalies are defined as deviations from monthly climatology calculated for the entire period.

### Detection of ENSO events

Detection of ENSO events (strong El Niño, single-year La Niña, and multi-year La Niña) is based on the time series of SST anomalies averaged in the Niño 3.4 region (170° W–120° W, 5° S–5° N) called N3.4. El Niño is defined when the ONDJF-mean value is above 0.5 K, and is categorized to strong El Niño when it exceeds 1.5 standard deviation. La Niña is defined when the ONDJF-mean value of N3.4 is below − 0.5 K, and is categorized to multi-year event when La Niña persists for two consecutive years. We did not analyze the 2015/16 strong El Niño event because the observational record ends in December 2016. We extracted six multi-year La Niña (1970, 1973, 1983, 1998, 2007, and 2010 as Year 0), four single-year La Niña (1964, 1988, 1995, and 2005), and six strong El Niño (1965, 1972, 1982, 1991, 1997, and 2009) events for the period 1961–2016 (Fig. S1). We confirmed that the identical sets of events were obtained using the Ocean Niño Index provided by NOAA (https://origin.cpc.ncep.noaa.gov/products/analysis_monitoring/ensostuff/ONI_v5.php), and also that all La Niña events occurred after El Niño in the previous year. Despite a difference in the SST data, extracted events are consistent with past multi-year La Niña studies^27,50^. When multi-year La Niña lasted for three years, we analyzed the first two years. For the CMIP6 multi-model analysis, the ENSO amplitude varies across models and therefore we used a threshold of 0.7 standard deviation to detect El Niño/La Niña.

### Ocean heat budgets

To quantify the processes that give rise to a large anomaly in OHC_eq_ during multi-year La Niña, we calculated ocean heat budgets for the equatorial area where OHC_eq_ was defined (120° E–60° W, 5.5° S–5.5° N and from the surface to 500 m). The selection of meridional boundary is based on meridional tilting mode of OHC integrated from the surface to 500 m (Fig. S9b, see supplementary Method).

1 $$\frac{\partial H}{{\partial t}} = \underbrace {{ - \mathop \int \limits_{{5.5^\circ {\text{N}}}} \rho C_{p} T \cdot v dS + \mathop \int \limits_{{5.5^\circ {\text{S}}}} \rho C_{p} T \cdot v dS}}_{{{\text{Recharge }}\;{\text{rate}}}} + \mathop \int \limits_{{120^\circ {\text{E}}}} \rho C_{p} T \cdot u dS + \mathop \int \limits_{{500{\text{m}}}} \rho C_{p} T \cdot w dS + \mathop \int \limits_{surface} Q dS,$$

where *H* indicates OHC_eq_; *T* is ocean temperature; and *u*, *v*, and *w* are the ocean zonal, meridional, and vertical currents, respectively. $\rho$ and *C*_*p*_ are the seawater density and specific heat of the ocean, respectively. *Q* is the net surface heat flux. *S* is the cross-sectional area of each boundary. The terms on the RHS represent ocean heat transport across boundaries, defining inward positive. The sum of the first two terms in Eq. (1) indicates heat exchanges across meridional boundaries, defining the recharge rate (i.e., Sverdrup heat transport). The third and fourth terms represent the heat exchange across the western and bottom boundaries, and the last term is the net heat gain/loss at the ocean surface. We used monthly mean data from the atmosphere and ocean reanalysis datasets for calculating the budget terms and confirmed that the sum of the RHS terms matches with the OHC_eq_ tendency well (Fig. S2). The OHC_eq_ defined in this study is highly correlated with the warm water volume in the equatorial Pacific ($r = 0.92$)^10^.

The recharge rate can be decomposed into contributions from surface Ekman current heat transport (EHT) and geostrophic current heat transport (GHT). We estimated EHT following the Ekman theory^51^ as

2 $${\text{EHT}} = - C_{p} \mathop \int \limits_{0}^{L} \frac{{\tau_{x} }}{f} T_{E} dx,$$

where $\tau_{x}$ is the zonal wind stress. *f* is the Coriolis parameter, and *L* is the basin width. The mean velocity-weighted temperature over the Ekman layer, *T*_*E*_, is calculated as

3 $$T_{E} = \sum v_{n} \left( z \right)T\left( {x,y,z} \right),$$

4 $$v_{n} \propto e^{z/\delta } {\text{sin}}\left( {\frac{ - z}{\delta } + \frac{\pi }{4}} \right),$$

where $v_{n}$ is a normalized velocity weight as a function of ocean depth, *z*. The Ekman layer depth, $\delta$, is assumed to be constant at 70 m, which is not sensitive to the different values, such as 50 or 100 m. The contribution from GHT is the difference between the recharge rate and the EHT term.

### Diagnosing steady atmospheric response to an imposed heating

We diagnosed a thermally forced steady atmospheric response using a linear baroclinic model (LBM)^52^. The model has a horizontal resolution of T42 and 20 vertical levels. The basic states, FMAMJ climatology, and anomalous diabatic heating (including condensational heating, radiative heating, surface heat flux, and subgrid-scale heat flux convergences)^53^ were calculated using the combined data of ERA40 and ERA-Interim. For simplification, the thermal forcing was confined to the tropical Pacific (120° E–80° W, 30° S–30° N).

### Statistical significance

We applied a two-sided Student’s t-test for the composite and correlation analyses, and Welch’s t-test for the difference between multi-year and single-year composite means. The confidence levels are described in the figure captions.

## Supplementary Information

Supplementary Information.

## Data Availability

The observation data, COBESST2 and PREC, are available at https://psl.noaa.gov/data/gridded/data.cobe2.html and https://psl.noaa.gov/data/gridded/data.precl.html. The ORAS4 and ORAS5 datasets are available at ftp://ftp-icdc.cen.uni-hamburg.de/EASYInit/ORA-S4/ and https://icdc.cen.uni-hamburg.de/thredds/catalog/ftpthredds/EASYInit/oras5/catalog.html; GECCO2 at ftp://ftp-icdc.cen.uni-hamburg.de/EASYInit/GECCO2/; GFDLECDAv3.1 at ftp://nomads.gfdl.noaa.gov/2/ECDA/ecda/GFDL-CM2.1-ECDA/; ERA40 at https://apps.ecmwf.int/datasets/data/era40-moda/levtype=sfc/; ERA-Interim at https://apps.ecmwf.int/datasets/data/interim-full-moda/levtype=sfc/. The CMIP6 datasets are available at https://pcmdi.llnl.gov/CMIP6/.

## Code availability

The analysis codes for this study are available from the corresponding author on reasonable request.
