# Supplementary material for: Mechanisms linking multi-year La Niña with preceding strong El Niño
Source: Sci Rep. 2021 Aug 25;11:17465. doi: 10.1038/s41598-021-96056-6 (PMC8387398; doi:10.1038/s41598-021-96056-6)
Supplement: Supplementary file 1 — Supplementary Information. [file 41598_2021_96056_MOESM1_ESM.pdf]

Supplementary Information

Mechanisms linking multi-year La Niña with preceding strong El Niño

Tomoki Iwakiri[1,*] and Masahiro Watanabe[1]

[1]Atmosphere and Ocean Research Institute, The University of Tokyo

*Corresponding author: Tomoki Iwakiri (iwakiri@aori.u-tokyo.ac.jp)
5-1-5 Kashiwanoha, Kashiwa, Chiba 277-8568, Japan

Supplementary method

Definition of the combination mode (C-mode)

To extract the C-mode, we conducted multi-variate empirical orthogonal function (EOF) analyses to combined anomalous zonal and meridional wind stress data over the tropical Pacific (120°–280° E, 10° S–10° N) following Stuecker et al[1]. The spatial patterns of the EOFs are presented by regressing the zonal and wind stress anomalies onto the corresponding principle component time series. The leading two EOFs, accounting for 21.6% and 14.8% of the total variance, represent the ENSO mature anomalies and the C-mode, respectively (Supplementary Fig. S4). The role of the C-mode in linking strong El Niño with multi-year La Niña is explained in the text.

Boundary selection for calculation of OHC_{eq}

To determine Pacific box for calculating ocean heat transport (OHC_{eq}), we conducted EOF analyses to anomalous OHC integrated from the surface to 500m over the tropical Pacific (120°–280° E, 30° S–30° N). In the ORAS4 for the period from 1961-2016, the leading two EOFs represent the east-west tilting mode and north-south tilting mode[2] (Fig. S9). We used north-south tilting mode to determine meridional boundary. In this study, we selected an axis along 5.5°N/S in reanalysis. Although southern boundary is not uniquely decided, the result is not sensitive because northern boundary primary contributes recharge/discharge mass transport. We repeatedly conducted this analysis for CMIP6 multi-model ensemble, and boundary we used summarized in Table S2.

Supplementary figures

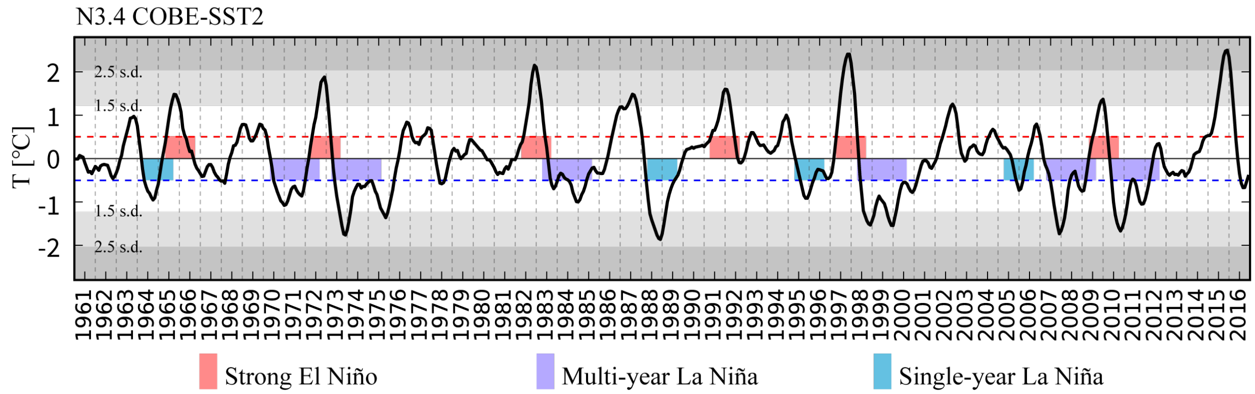

Supplementary Figure S1. Observed time series of N3.4. Black line indicates observed time series of N3.4 from COBE-SST2. Time series is smoothed with a three-month running mean filter. Red and blue dashed lines denote 0.5 K and -0.5 K, respectively. Bars indicate individual ENSO events classified into extreme El Niño, multi-year La Niña, and single-year La Niña.

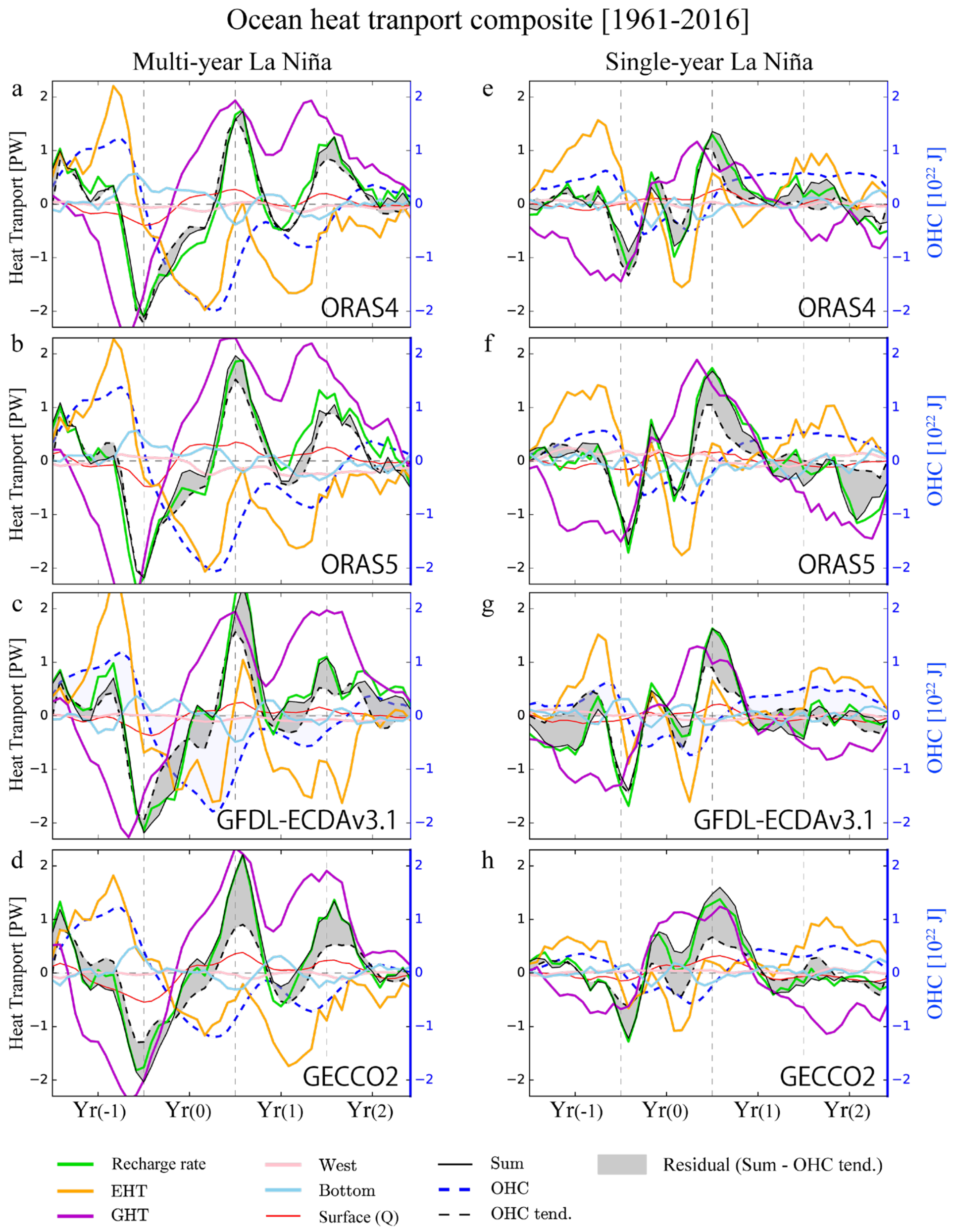

Supplementary Figure S2. Ocean heat transport composites in the equatorial Pacific box among four ocean reanalysis datasets. (**a**) ORAS4, (**b**) ORAS5, (**c**) GFDL-ECDAv3.1, and (**d**) GECCO2 for multi-year La Niña. (**e-h**) As in (a-d) but for single-year La Niña.

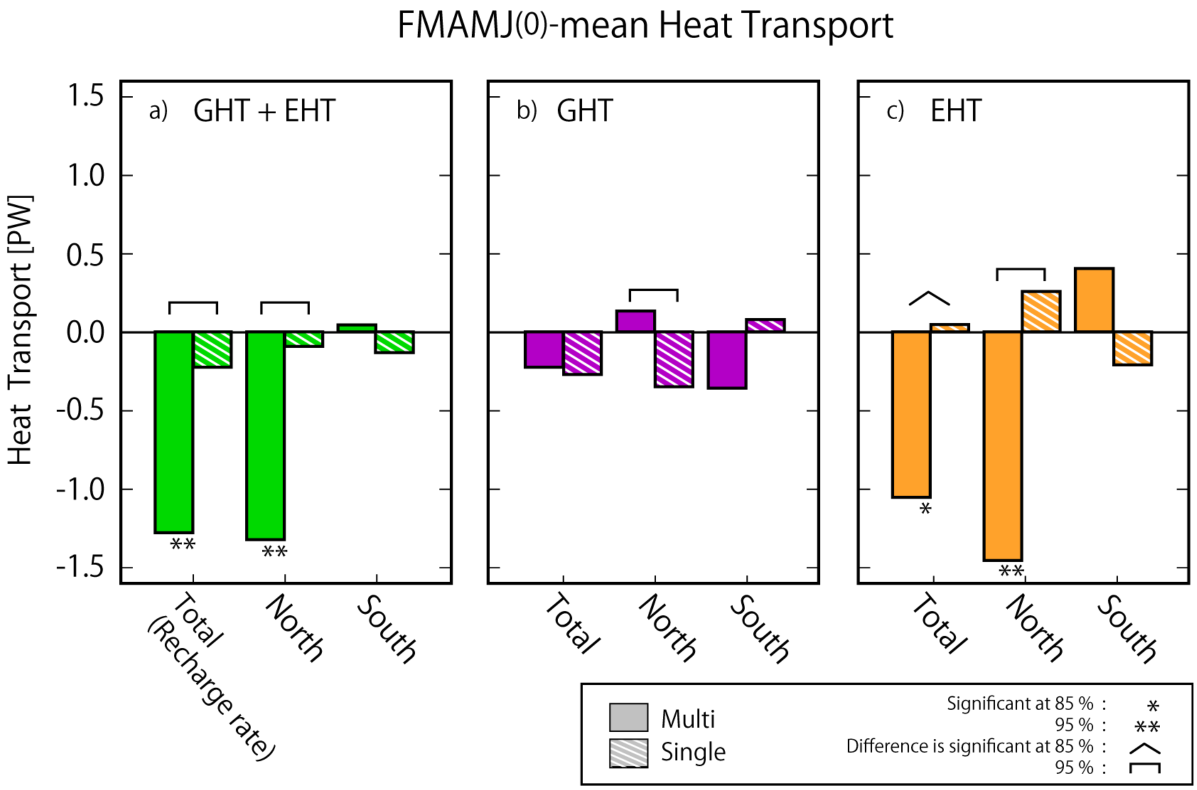

Supplementary Figure S3. FMAMJ(0)-mean ocean heat transport across north and south boundaries of the equatorial Pacific box. (**a**) Recharge rate equivalent to the sum of EHT and GHT, (**b**) GHT, and (**c**) EHT. Solid (hatched) bars are the composites for multi-year (single-year) La Niña. In each panel, total heat transport and contributions at the northern and southern boundaries are shown from left to right. Asterisks and brackets represent the level of statistical significance, as explained in the figure legend.

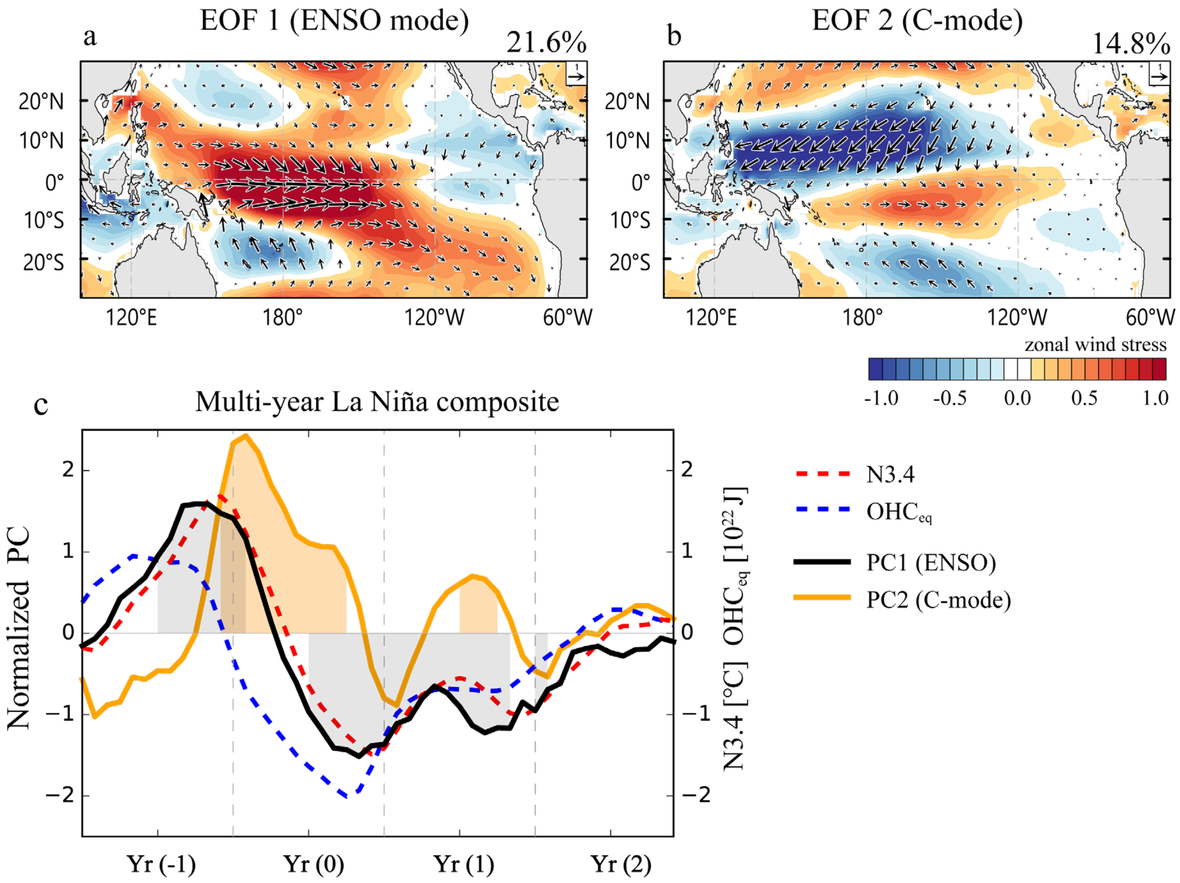

Supplementary Figure S4. Combination mode (C-mode) and multi-year La Niña. (**a,b**) First and second EOF patterns of wind stresses (units in N/m^2). Shading indicates the zonal wind stress. (**c**) multi-year La Niña composite of PC1 (black), PC2 (orange), N3.4 (red dashed line), and OHC_{eq} anomaly (blue dashed line). Shadings represent that the composite PC anomalies are statistically significant at the 95% confidence level.

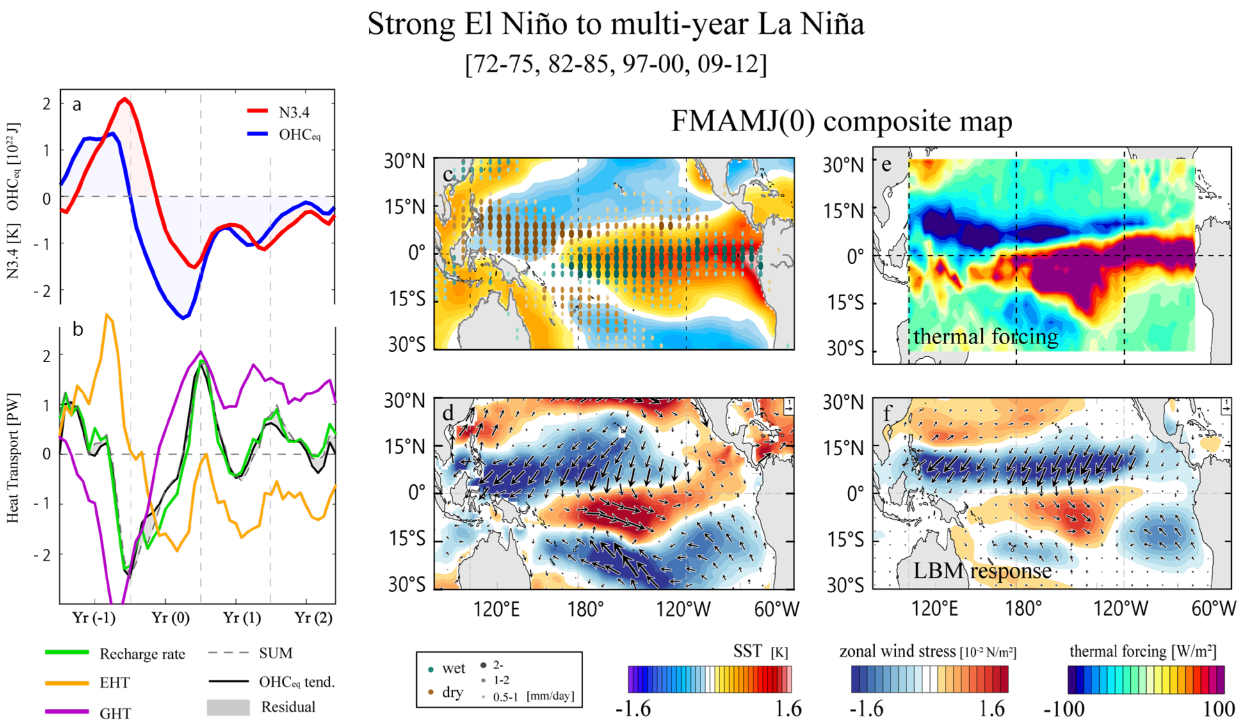

Supplementary Figure S5. Composite time-series and maps for the strong El Niño to multi-year La Niña case. (a,b) composite time-series of N3.4 (red), OHC_{eq} (blue), recharge rate (green), GHT (purple), EHT (orange), SUM (dashed line), OHC_{eq} tendency (black), and residual (shading) for strong El Niño to multi-year La Niña (four events mean). (**c**) Anomalous SST (shading) and precipitation (dots) and (**d**) surface zonal wind stress (shading) and wind stress vector (units in 10^{-2} N/m^2) in FMAMJ(0). (**e,f**) As seen in Fig. 4.

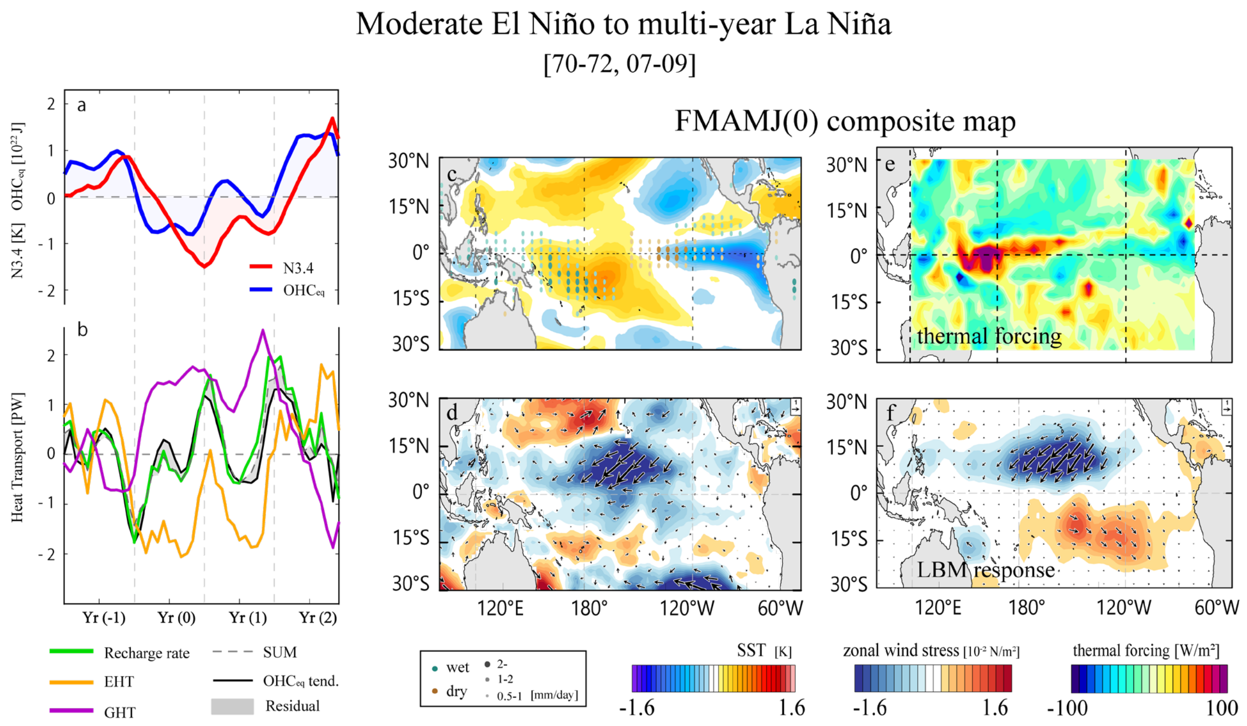

Supplementary Figure S6. Composite time-series and maps for the moderate El Niño to multi-year La Niña case. (a-f) As in Fig. S6 but for the moderate El Niño to multi-year La Niña case (two events mean).

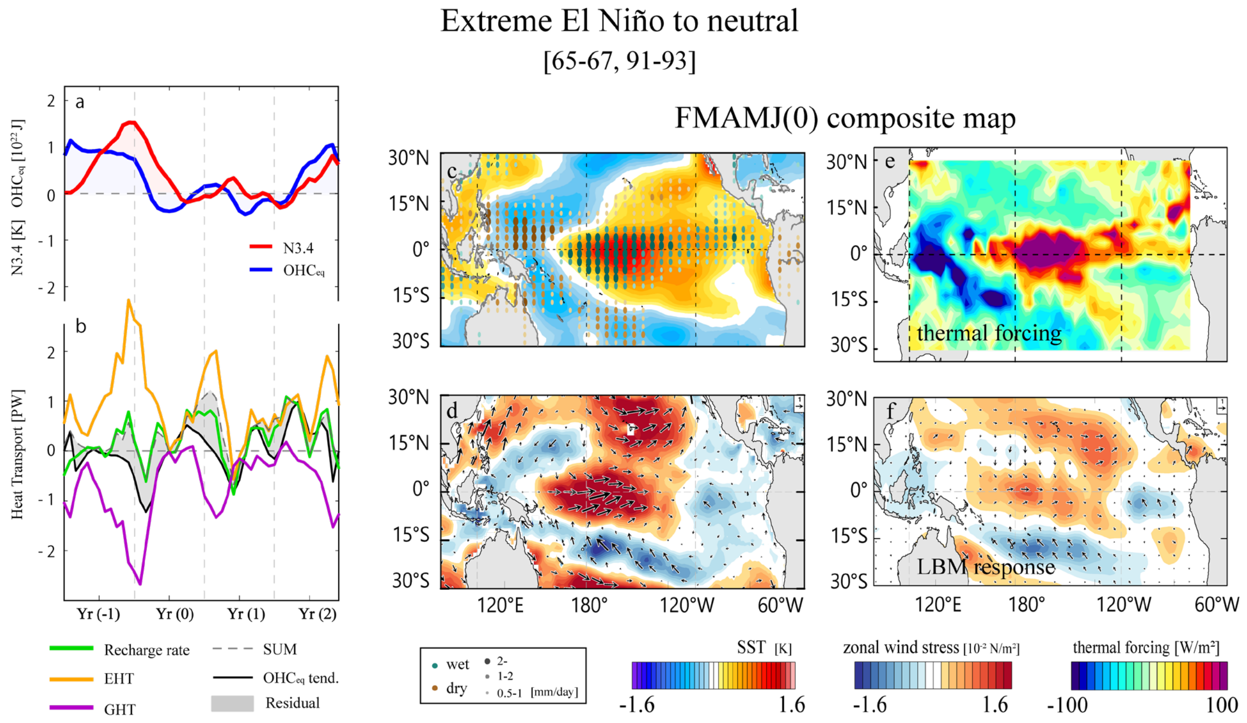

Supplementary Figure S7. Composite time-series and maps for the extreme El Niño to neutral case.

(a-f) As in Fig. S6 but for the extreme El Niño to neutral case (two events mean).

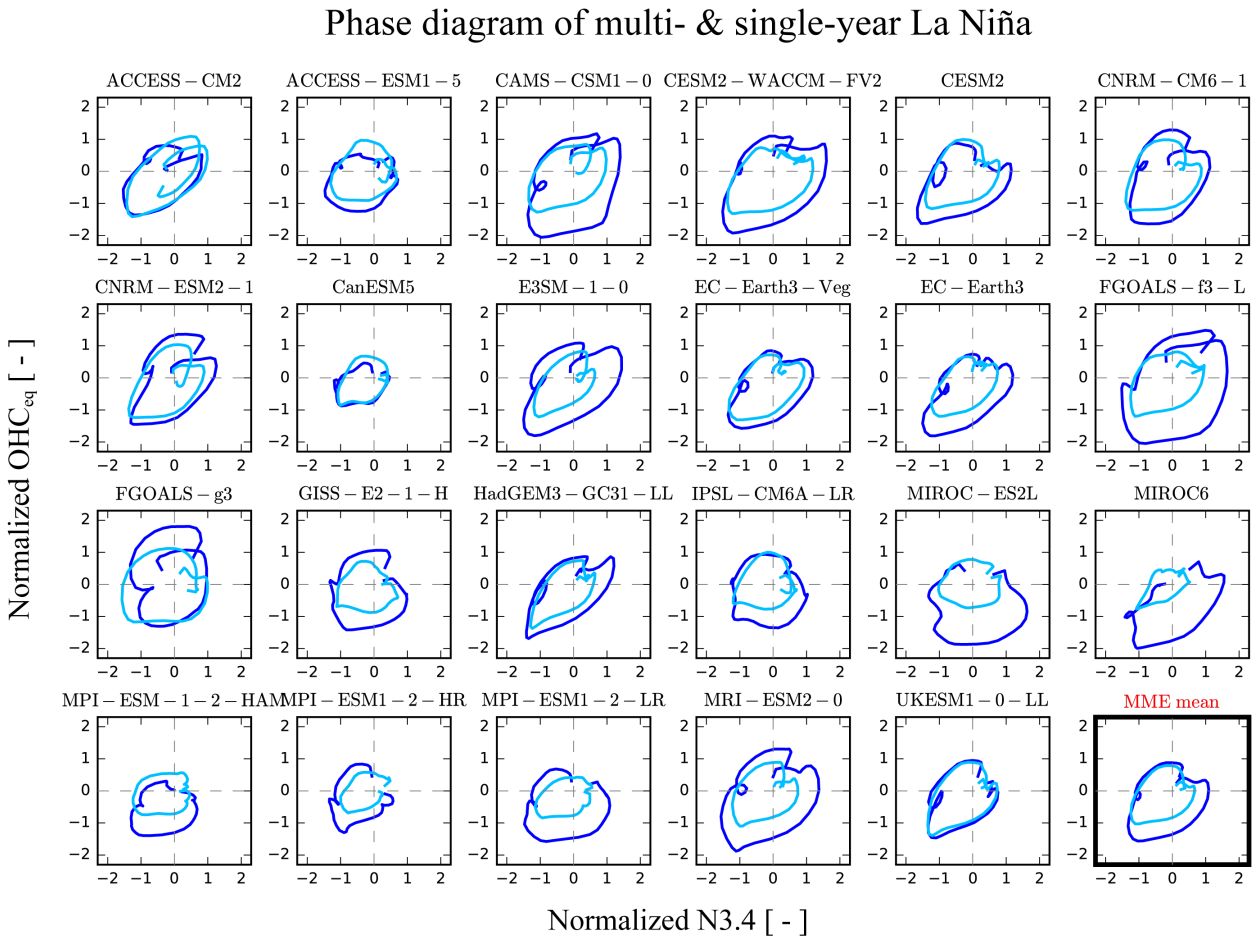

Supplementary Figure S8. As in Figure 1a but for each CMIP6 model and multi model ensemble (MME) mean. Horizontal and vertical axes are normalized.

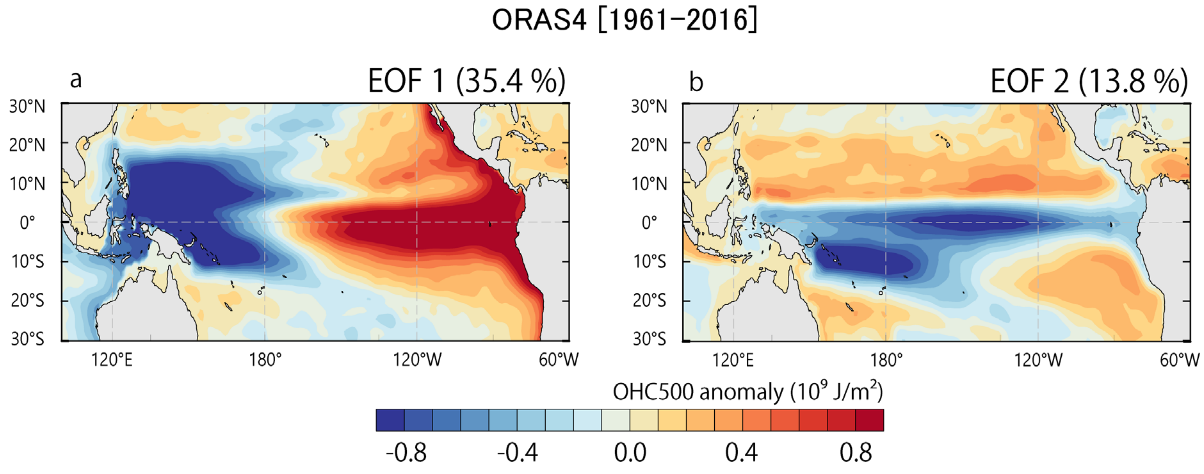

Supplementary Figure S9. Zonal and meridional tilting mode of OHC. (a,b) First and second EOF mode of anomalous OHC integrated from the surface to 500m.

Supplementary Table S1. N3.4 and GHT-induced recharge rate during NDJ(-1/0).

NDJ(-1/0)	Multi-year	Single-year
N3.4 [K]	1.68	0.94
GHT [PW]	-2.13	-1.31
Normalized GHT [PW/K]	-1.27	-1.39

Supplementary Table S2. 24 CMIP6 models used in the present study and observational SST data. Number of ENSO events and meridional boundary to calculate OHC_{eq} are also summarized.

Symbol	Model/Data	Period	#Number of		boundary	
			Multi La Niña	Single La Niña	South (°S)	North (°N)
	CMIP6 models	(500 years)				
**A**	ACCESS-CM2	950-1449	9	119	10.0	6.0
**B**	ACCESS-ESM1-5	101-600	18	98	10.0	8.5
**C**	CAMS-CSM1-0	2900-3390	18	109	12.5	7.5
**D**	CESM2-WACCM-FV2	1-500	35	81	7.5	8.5
**E**	CESM2	1-500	29	75	7.5	7.5
**F**	CNRM-CM6-1	1850-2349	18	91	9.0	7.5
**G**	CNRM-ESM2-1	1850-2349	21	100	10.0	8.5
**H**	CanESM5	5201-5700	24	66	10.0	10.0
**I**	E3SM-1-0	1-500	30	76	10.0	8.5
**J**	EC-Earth3-Veg	1850-2349	29	88	8.5	8.5
**K**	EC-Earth3	2259-2758	29	73	8.5	8.5
**L**	FGOALS-f3-L	600-1099	27	94	8.5	6.5
**M**	FGOALS-g3	200-699	4	128	10.0	5.5
**N**	GISS-E2-1-H	3180-3679	30	67	10.0	10.0
**O**	HadGEM3-GC31-LL	1850-2349	30	72	7.5	7.5
**P**	IPSL-CM6A-LR	1850-2349	22	89	10.0	10.0
**Q**	MIROC-ES2L	1850-2349	47	34	10.0	9.0
**R**	MIROC6	3200-3699	36	39	8.0	9.0
**S**	MPI-ESM-1-2-HAM	1850-2349	26	69	9.0	6.5
**T**	MPI-ESM1-2-HR	1850-2349	36	43	13.5	9.0
**U**	MPI-ESM1-2-LR	1850-2349	39	44	10.0	8.5
**V**	MRI-ESM2-0	1850-2349	32	76	12.0	10.0
**W**	UKESM1-0-LL	1960-2459	30	65	7.5	9.0
	Total		619	1796		
	Observations					
	COBE-SST2	1961-2016	6	4	5.5	5.5
	COBE-SST2	1901-2018	10	11	5.5	5.5

Supplementary References

1. Stuecker, M. F., Timmermann, A., Jin, F.-F., McGregor, S. & Ren, H.-L. A combination mode of the annual cycle and the El Niño/Southern Oscillation. *Nature Geoscience* **6**, 540–544 (2013).
2. Meinen, C. S. & McPhaden, M. J. Observations of warm water volume changes in the equatorial Pacific and their relationship to El Niño to La Niña. *Journal of Climate* **13**, 9 (2000).
